# A Novel Parametrical Approach to the Ribbed Element Slicing Process in Robotic Additive Manufacturing

**DOI:** 10.3390/polym17141965

**Published:** 2025-07-17

**Authors:** Ivan Gajdoš, Łukasz Sobaszek, Pavol Štefčák, Jozef Varga, Ján Slota

**Affiliations:** 1 Department of Technology, Materials and CAx, Faculty of Mechanical Engineering, Technical University of Košice, 04001 Košice, Slovakia; ivan.gajdos@tuke.sk (I.G.); pavol.stefcak@tuke.sk (P.Š.); jan.slota@tuke.sk (J.S.); 2 Department of Information Technology, Faculty of Mathematics and Information Technology, Lublin University of Technology, 20-618 Lublin, Poland; 3 Prototyping and Innovation Centre, Faculty of Mechanical Engineering, Technical University of Košice, 04200 Košice, Slovakia; jozef.varga.2@tuke.sk

**Keywords:** additive manufacturing, industrial robot, slicer, robotic 3D printing

## Abstract

Additive manufacturing is one of the most common technologies used in prototyping and manufacturing usable parts. Currently, industrial robots are also increasingly being used to carry out this process. This is due to a robot’s capability to fabricate components with structural configurations that are unattainable using conventional 3D printers. The number of degrees of freedom of the robot, combined with its working range and precision, allows the construction of parts with greater dimensions and better strength in comparison to conventional 3D printing. However, the implementation of a robot into the 3D printing process requires the development of novel solutions to streamline and facilitate the prototyping and manufacturing processes. This work focuses on the need to develop new slicing methods for robotic additive manufacturing. A solution for alternative control code generation without external slicer utilization is presented. The implementation of the proposed method enables a reduction of over 80% in the time required to generate new G-code, significantly outperforming traditional approaches. The paper presents a novel approach to the slicing process in robotic additive manufacturing that is adopted for the fused granular fabrication process using thermoplastic polymers.

## 1. Introduction

Currently, 3D printers are being utilized for prototyping and developing structures. The appeal of this solution mostly stems from its capability to facilitate the additive manufacturing (AM) of three-dimensional items featuring intricate geometries [1]. The topic of additive manufacturing usually evokes associations with 3D printing (3DP) conducted by means of devices executing the process using the Cartesian coordinate system, where the nozzle is always perpendicular to the working surface and only moves parallel to it [2]. In this area, researchers have proposed various solutions in the areas of device construction, components, and software [3,4,5,6,7]. However, an analysis of the capabilities of commonly used equipment employed in the manufacturing process reveals certain limitations regarding the structure of the fabricated components. These limitations are primarily associated with the position and orientation of the extruder [8,9].

**Figure 1:**
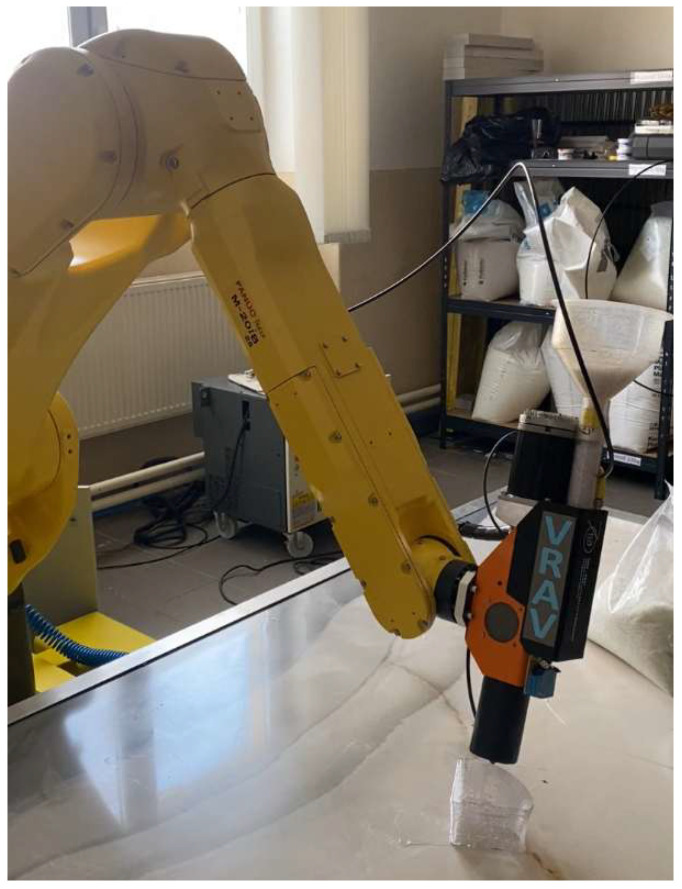
Robotic AM example.

The development and market adoption of 3D printing technology have opened up opportunities for the use of industrial robots with multiple degrees of freedom in additive manufacturing processes [10,11]. Multi-axis movement of the printing module in 3DP enables the production of more complex geometries and enhances precision. The progression of additive manufacturing (AM) technology has enabled the incorporation of industrial robots featuring multiple degrees of freedom into the fabrication process [12,13,14]. The multi-axis movement of the printing module in robotic 3DP enables the production of more complex geometries and enhances precision (Figure 1).

A crucial step in the AM process is the division of the model into layers, commonly referred to as ‘slicing’ [13]. However, the well-known solutions in this area are mainly dedicated to classic 3D printers and therefore do not fully support the process of robotic additive manufacturing [15,16,17]. Consequently, this process necessitates the use of various software, thereby impacting its overall duration. In response to the problem noted, a novel solution is presented in this article, which employs a parametric model of the prototyped and manufactured component to directly generate control code for an industrial robot in the 3DP process.

In Section 2, methodologies related to robotic additive manufacturing (AM) are discussed, with a specific focus on the slicing stage of the process. Section 3 presents a description and the assumptions of the proposed solution, dedicated to ribbed-element additive manufacturing. Section 4 describes experimental verification of the developed system. The proposed approach was validated through its application in the fabrication of frameworks and thin mats, as well as in the reconstruction of machine components. The paper is concluded with a sub-summary and plans for further research.

## 2. Existing Work Discussion

### 2.1. Robots in Additive Manufacturing

Observing the limitations of the devices utilized in the 3DP process executed in the Cartesian coordinate system, industrial robots with an extruder installed began to be proposed for use in the AM process [13,16,18]. The main benefit of this approach is the enhanced kinematic flexibility and substantial workspace of industrial robots, allowing for the production of parts with increased dimensions [19], different structures [4], and no need to build support structures [20]. An additional advantage is that industrial robots are equipped with advanced control systems, which facilitate their integration and operation within the 3D printing process [5,18]. However, in order to use the robot in the mentioned process, the robot must be properly adapted to the requirements of the AM technology. It is essential to appropriately select the devices and actuators, ensure their proper physical integration with the robot, and establish suitable control systems and IT tools to enable precise execution of the robot’s movements [16,21].

Robotic 3D printing is a subject of considerable interest and has been widely discussed in numerous research publications. Authors have proposed various solutions and the use of different additive manufacturing techniques [4,22,23]. However, it should be noted that within the field of additive manufacturing, the use of industrial robots is most commonly associated with two widely adopted technologies: Fused deposition modeling (FDM) and fused filament fabrication (FFF) [5,22,24,25]. These technologies entail the application of numerous thin layers of molten plastic until the required structure is achieved. The processes involve the sequential deposition of thermoplastic material, typically in the form of a thin filament composed of materials such as ABS, PLA, or PET-G [2]. The low cost of manufacturing, the high strength of the parts, the potential for further mechanical processing, and water resistance are examples of advantages of products manufactured using FDM/FFF [26]. As robotic 3DP is very often concerned with the manufacture of large-scale components, a popular variation of these processes is screw-extrusion additive manufacturing (SEAM), in which the material is fed in the form of pellets [9].

In addition to the techniques previously mentioned, various alternative approaches and solutions have been proposed in the field of robotic 3D printing. For instance, ref. [26] describes a case study of programming industrial robots for 3DP of metal products. It should be noted that such implementations are becoming increasingly popular and widely used, as is the use of cementitious material or composite material 3DP [27]. Among the various approaches, concrete 3D printing has gained significant popularity, particularly in large-scale construction applications [28]. In this area, researchers have proposed a variety of solutions, driven by the unique characteristics of the building materials used [13,29,30]. An interesting solution was presented in ref. [31]. The authors describe a mobile robotic printer system that requires a range of collaborative solutions in view of robots carrying out the common 3DP process [12]. The continued development of the area is demonstrated by the novel solutions presented in the literature. An example of polygon mesh printing that is collision-free during robotic operation was presented in [32]. An additional noteworthy solution within the described approach is the utilization of collaborative robots.

As was mentioned, researchers not only analyze the overall processes and various technologies but also focus on their key phases. For example, a significant phase in additive manufacturing is the slicing process, which involves the division of the model into layers [33,34,35]. The literature highlights the absence of standardized software for controlling industrial robots used as FDM or FFF systems [5]. As a result, numerous studies have proposed various solutions to address this issue.

### 2.2. Key Role of Slicing Process

The problem of appropriate slicing software and solution development have been the subject of consideration by researchers [5,17,18,36]. The slicer constitutes a crucial element of robotic AM that allows the control of various characteristics of the additive manufacturing process—infill percent, layer thickness, type of infill pattern, and extrusion speed [37]. Based on the slicer result, a toolpath for the extruder to create each layer is generated. The tool is then converted to G-code commands that drive the device kinematic system [38]. In [35], it was emphasized that the layering process can directly affect the manufactured elements surface finish and mechanical properties. Ref. [23] investigates the impact of slicer selection on the printing process, and the problem was also noted in refs. [33]. Moreover, in [8], the eco-impacts and eco-efficiency of the layering process were emphasized.

In response to the key role of slicing, various solutions have been proposed. For example, a new algorithm to generate filled NP layers was proposed in [22], using a contouring method that reduces the layer thickness variation. An interesting solution was proposed in refs. [9,31], where a new slicing scheme and a slicer for cooperative 3DP with mobile robots working together on one print job were described. In ref. [39], a novel slicing strategy in robotic AM was presented that allows the manufacture of elements without support material. A hand motion-based 3DP slicer is proposed in turn in [40]. In [41], a parallel implementation of the slicing algorithm using a GPU was described. In certain approaches, the additive manufacturing process has been implemented without relying on traditional slicers, through the direct generation of toolpaths [3].

An important and frequently raised issue is dedicated software development that can support model layering and control codes generated in the process of robotic AM [5]. A mathematical algorithm to project a robot trajectory as a sequence of points on any triangle-tessellated non-planar surface was described in [42]. The authors of [19] presented a slicer for a direct extrusion system. In turn, a novel slicing algorithm for planar and non-planar trajectories was presented in [34]. In ref. [5], the authors presented a solution that allows the generation of code that can be executed directly on an ABB robot in the FFF process. An algorithm for non-planar path planning with variable layer height implemented in Java was described in [36]. In [1], a method for medical-model slicing was presented and compared with the conventional STL-based method. The study presented in [43] introduces a slicing approach that involves the integration of two distinct software tools, each serving different functions, to accomplish effective layer generation.

It should be stated that most solutions proposed in the literature are based on the use of a variety of computer programs simultaneously. Identifying a suitable tool for the slicing process in the field of robotic AM has been researched in many works [8,18,21]. For example, ref. [16] describes a solution in which a robot’s end effector travels along the toolpath modeled by the Robot Studio software, and the base of the process is an STL file, which is divided into layers based on thickness by means of a MATLAB (R2018a) tool. An STL-based solution is also outlined in [5]. In ref. [4], the authors analyze the robotic printing of sustainable structures made from wood fiber polymer composites. To manufacture the element, several types of software were used: traditional CAD, Cura Slicer, and Robot Studio. In turn, in ref. [17], to conduct the robotic AM process, it was necessary to use a developed C# programming language tool for generating toolpaths and G-Code, which was implemented using the Rhino Common API. A practical slicing software implementation using the LabView-supplied Dynamic Linked Library was described in [18]. Similarly, in ref. [20], a very interesting but complex double-transformation method for robotic AM requiring the use of multiple tools was described. The use of the Robot Operating System (ROS) is occasionally proposed in the literature as a framework for managing robotic additive manufacturing processes. However, it is more common to utilize another software for layering processes—open source Slic3r [21]. The use of multiple software tools is often required, particularly in prototyping processes involving 3D printing. For instance, in ref. [11], robotic AM was implemented to develop a personalized insole surface. Nevertheless, to achieve the aim, it needed the use of numerous software programs: Ubuntu OS, Notepad++, MATLAB, Microsoft Excel, CAD software, and Slic3r. Similarly, in ref. [16], where a hybrid toolpath-generating method for six-axis-robot-integrated FDM processes was considered; different computer programs (CAD and slicing software, MATLAB, Robot Studio) were utilized.

This underscores the need for integrated solutions that streamline the robotic AM workflow by minimizing the reliance on multiple software tools (Table 1). A novel approach would certainly reduce model preparation time and facilitate the work of engineers. The solutions proposed in the literature mainly require the use of multiple tools, which translates into increased time to prepare the model for the manufacturing process. An innovative approach is presented in this paper, aiming to shorten the pre-processing time and enhance the slicing workflow for robotic additive manufacturing. By utilizing a parametric model of the printed component, the method streamlines standard procedures, minimizes the reliance on multiple software tools, and facilitates straightforward generation of control code for the printing robot. Currently, time reduction is one strategy for improving 3DP highlighted in the research [42]. Researchers have also outlined the problem in the literature [9,25,44]. It has been mentioned that the direct slicing method may be the solution, but the number of papers in this field is very limited [45]. In response to the problems noted in this paper, a novel approach to the slicing process dedicated to ribbed-element robotic AM is presented.

## 3. A Novel Parametrical Approach to the Slicing Process in Robotic Additive Manufacturing

### 3.1. Objectives

**Figure 2:**
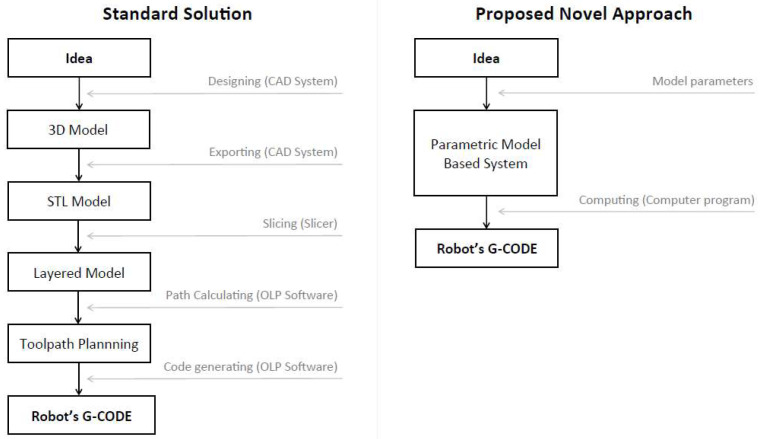
Comparison of typical solution and proposed novel approach in robotic 3DP.

As noted in Section 2.2, researchers in the field of robotic AM primarily utilize well-known slicers and additional software to prepare models for the robotic 3DP process. As a result, the overall manufacturing process necessitates the use of a variety of tools, consumes a significant amount of time, and has a limited ability to freely modify process parameters. However, the use of an industrial robot in the AM process allows numerous parameters to be modified precisely (such as the height of the printing layer or the speed of the robot arm). This indicates a clear need for the development of methods aimed at optimizing and simplifying the model preparation stage in robotic additive manufacturing workflows. In response to the identified challenges, we propose a novel methodological approach. The primary objectives of its development are to reduce model preparation time and minimize the number of software tools required. In Figure 2, the difference between the standard solution and the proposed novel approach is presented.

The proposed approach focuses on developing a system that streamlines the transition from part design to 3D printing robot control code generation. The solution responds to the problems observed and outlined in the literature, which indicate that multiple computer tools are required to conduct the robotic AM process. Consequently, there is a need for a variety of licenses (which in turn impose additional costs on users), extensive knowledge of alternative software programs, and the overall time required for component prototyping and designing is high. In response to the problems identified, a system based on a parametric model of the part to be designed is proposed. The novel approach enables efficient Robotic 3D printing by bypassing and accelerating conventional stages of the manufacturing workflow.

### 3.2. Model of the Proposed Solution

**Figure 3:**
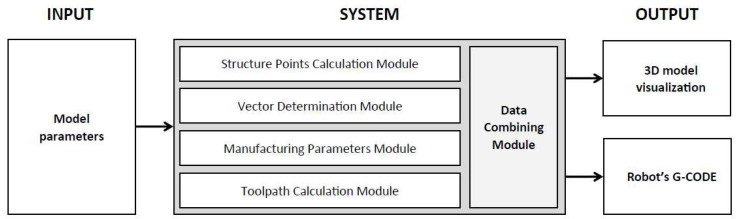
Parametrical model-based system scheme for robotic AM.

The proposed solution has the character of a multi-modular system developed for the robotic AM of ribbed parts. It is based on a new approach to the slicing process, which is conducted based on a mathematical model and a determined point sequence. This innovative solution is an alternative to the well-known slicing process based on the STL model. The input to the system is a parametric description of the component to be manufactured, while the output comprises the robot control code and a visualization of the corresponding toolpaths. The system comprises appropriately designed modules that transform and combine the input data in stages to generate the output results. A visualization of the system is shown in Figure 3.

The key elements of the innovative approach being developed include the following:

- The Structure Points Calculation Module uses the basic geometrical parameters that describe the manufactured component and determines the basic point coordinates.
- The Vector Determination Module is responsible for the definition of geometrical data relations and vector calculations.
- The Toolpath Calculation Module allows for toolpath direction and changes to it based on the vectors generated.
- The Manufacturing Parameters Module is responsible for defining the robotic 3DP process parameters for the individual manufacturing steps.
- The Data Combining Module combines data resulting from the use of the system modules and presents it in the form of G-Code.

The module functionalities were designed to be implemented using freely available software tools and high-level programming languages.

**Figure 4:**
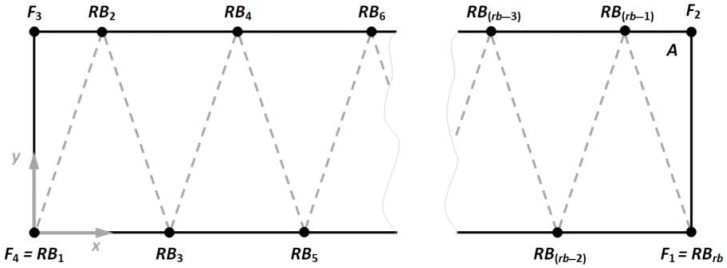
Assumed structure of manufactured element base layer.

The basis for the proposed system utilization is an adequate parametric description of the ribbed element to be manufactured. Assuming that: a—structure length, b—structure width, rb—number of ribs the shape of the base layer (rb∈2N), understood as the set of points shown in Figure 4, can be defined. Moreover, assumed that h—structure height, $L_{h}$—layer height, $L_{q}$—layers quantity, CL—current layer, $V_{rob}$—robot velocity, $T_{noz}$—nozzle temperature, the robots control parameters can be defined accordingly.

Based on the assumed parametric model, the coordinates of key points of the base layer of the manufactured structure can be determined:

(1) $$F_{1}={RB}_{rb}=\left(a,0\right),\;F_{2}=\left(a,b\right),\;F_{3}=\left(0,b\right),\;F_{4}={RB}_{1}=\left(0,0\right),$$

(2) $${RB}_{i}=\left(k\cdot \frac{2a}{rb},0\right),i=\left\{3,\ldots ,rb-2\right\}\cup i\in 2N+1,k=\left\{1,\ldots ,\frac{rb}{2}-1\right\},$$

(3) $${RB}_{j}=\left(\frac{a}{rb}+l\cdot \frac{2a}{rb},b\right),j=\left\{2,\ldots ,rb-1\right\}\cup j\in 2N,l\in \left\{0,\ldots ,\frac{rb}{2}-1\right\}$$

In turn, the definition of the mentioned points allows the determination of the vectors necessary for the subsequent generation of the paths of a robot. Accordingly, the vector values can be defined as follows:

(4) $$\left|\vec{F_{1}F_{2}}\right|=\left|\vec{F_{3}F_{4}}\right|=b,$$

(5) $$\left|\vec{F_{2}F_{3}}\right|=\left|\vec{F_{4}F_{1}}\right|=a,$$

(6) $$\left|\vec{{RB}_{1}{RB}_{2}}\right|=\left|\vec{{RB}_{2}{RB}_{3}}\right|=\ldots =\left|\vec{{RB}_{\left(rb-1\right)}{RB}_{rb}}\right|=\sqrt[{2}]{b^{2}+{\left(\frac{a}{rb}\right)}^{2}},rb\in 2N$$

Utilizing the values of the parameters h and $L_{h}$, it is possible to determine the quantity of layers $L_{q}$ (iterations) that should be executed by the robot to fabricate the structure:

(7) $$L_{q}=\frac{h}{L_{h}}$$

Then the set $CL=\{1,\ldots ,\;L_{q}\}$ can be defined, which in turn allows the determination of the vector sequence forming the path of the robot tool:

(8) $$\vec{F_{1}F_{2}},\vec{F_{2}F_{3}},\vec{F_{3}F_{4}},\vec{F_{4}{RB}_{rb}},\vec{{RB}_{1}{RB}_{2}},\vec{{RB}_{2}{RB}_{3}},\ldots ,\vec{{RB}_{\left(rb-1\right)}{RB}_{rb}},forCL\in 2N+1$$

(9) $$\vec{F_{4}F_{3}},\vec{F_{3}F_{2}},\vec{F_{2}F_{1}},\vec{F_{1}{RB}_{rb}},\vec{{RB}_{1}{RB}_{2}},\vec{{RB}_{2}{RB}_{3}},\ldots ,\vec{{RB}_{\left(rb-1\right)}{RB}_{rb}},forCL\in 2N$$

**Figure 5:**
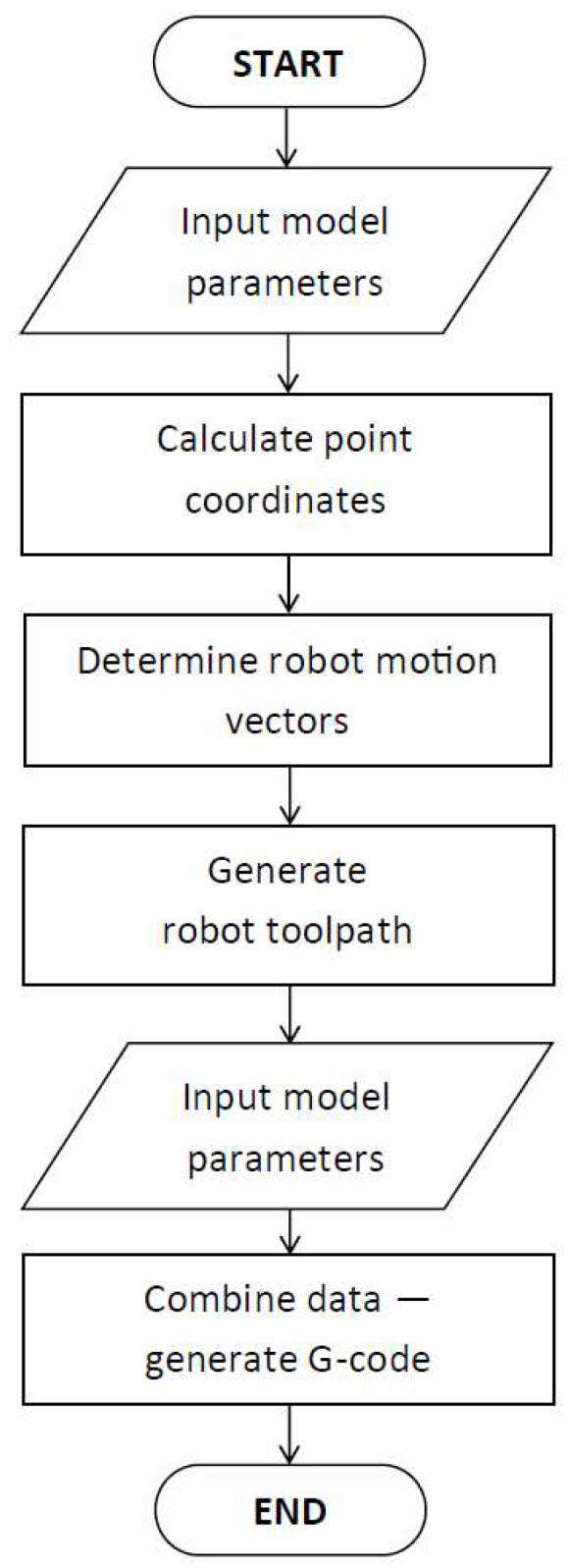
Proposed algorithm for a novel robotic 3D printing approach.

The data obtained is converted into G-Code, which is then expanded with key process parameters defined by the operator (robot speed at individual points, extrusion values). Figure 5 shows the general algorithm of the proposed solution.

The proposed approach’s functionalities were designed for implementation using freely available software tools and high-level programming languages. Based on standard programming constructs (e.g., conditional statements), the solution can be implemented using any software capable of coding, data analysis, and processing (e.g., MATLAB or Python).

### 3.3. Experimental Verification

**Figure 6:**
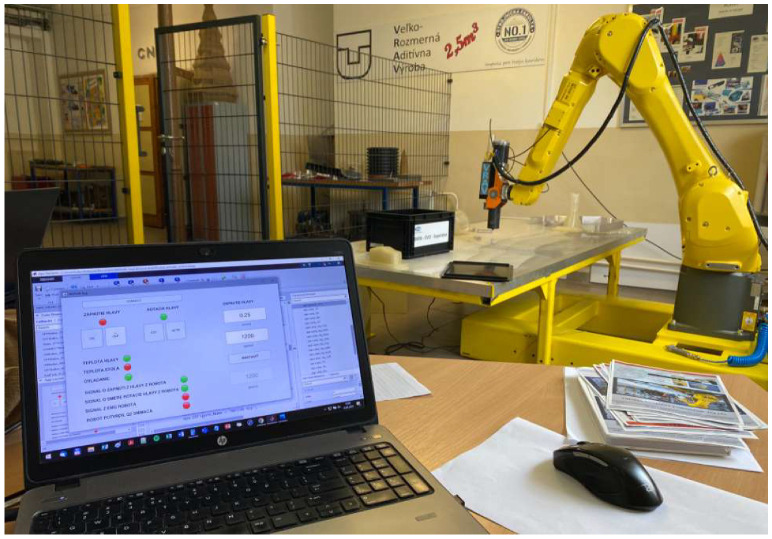
The utilized robotic 3DP cell.

To verify the proposed solution, appropriate tests were carried out. All of the presented work was conducted by means of a constructed robotic AM workstation (Figure 6) equipped with an FANUC M-20iB robot, a Massive Dimension MDPH2 pellet head extruder, an Industrial Shields M-Duino 57R I/Os PLUS PLC, and a heating bed. The utilized printing technology was screw-extrusion additive manufacturing (SEAM), which is similar to FDM. The only difference is the form of the printing material supplied. In the case of FDM technology it is a filament, while in the case of SEAM it is pelletized plastic.

**Figure 7:**
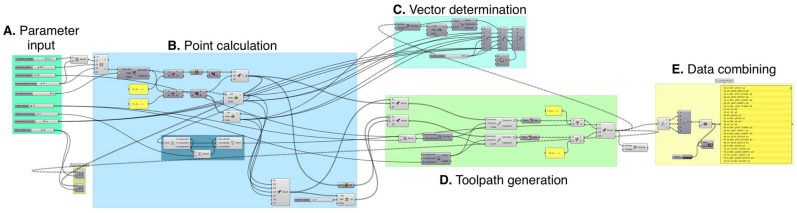
Part of the graphical program responsible for the calculation and its conversion into command form.

To verify the proposed novel approach in the process of robotic AM of ribbed components, implementation of the solution as a computer-based program was necessary. For this purpose, the Rhinoceros software with the Grasshopper 3D module for graphical programming was used (Figure 7). The ability to implement geometric relationships, arithmetic calculations, as well as use conditional instructions and functions for converting data into text form, made it possible to verify the correctness of the proposed solution.

The implementation of the proposed solution was structured into dedicated functional blocks to streamline the execution of the methodology presented in the article. Section A utilizes standard input components, where parameter values are defined through slider-based controls. Section B incorporates function blocks, responsible for data aggregation, computational operations, and geometric transformations. List management commands are employed to organize and pre-process the data used in section C. The output of these list operations is further utilized in section D, where the data are transformed into vector representations and integrated into a complete robotic toolpath. In section E, model parameters are combined with robot operation parameters using summation blocks, enabling the generation and listing of the resulting control code.

The aim of the research was the manufacturing of ribbed frameworks of alternative sizes. Furthermore, in order to verify the possibility of modifying the parameters, the process was carried out for alternative structures and with different construction materials. Another important consideration was the method’s practical implementation. Therefore, a reconstruction of the reed component from a shuttleless loom was performed as a case study.

### 3.4. Verification of Proposed Approach

The first part in the process of verifying the process of ribbed-element slicing was to generate models with the use of the developed computer program. For this aim, various dimensions and parameters of the manufactured parts were defined, observing the output results (whether the objects are visualized correctly, and the generated code is correct). The results of the tests carried out are shown in Table 2. The tests carried out proved the effectiveness of the solution. Consequently, the next stage of the research was conducted.

**Figure 8:**
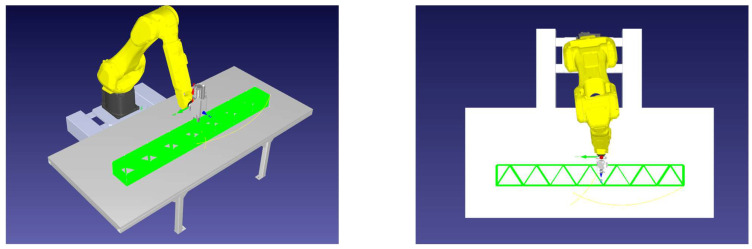
Developed system output G-Code testing in the RoboDK™ software.

The next stage of the research involved simulating a robot carrying out additive manufacturing processes in a virtual robot development environment. The RoboDK™ offline programming software (OLP) was utilized to validate the properties of the developed control codes (Figure 8). Based on the built-in “3D Printing Project” tool utilized, it was possible to implement the generated G-Code and observe the robot’s operation. The process was aimed at checking the movements of the robot tool in the individual layers of the manufactured part (according to the vectors and assumptions made in the mathematical model).

**Figure 9:**
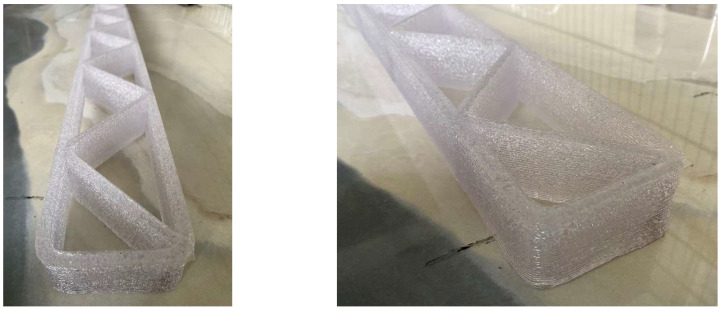
Ribbed framework manufactured using the proposed solution.

The positive results of the manufacturing simulation process in an OLP environment allowed robotic 3DP of the ribbed framework to be carried out. The process included the fabrication of a large-sized component (Figure 9) with dimensions a = 1600 [mm], b = 100 [mm], h = 40.5 [mm]; as well as smaller components (Figure 9) with dimensions a = 222 [mm], b = 50 [mm], h = 37.5 [mm], and $L_{h}$ = 2 [mm]. The robot control parameters were $V_{rob}\;$= 20 [mm/s] and $T_{noz}$ = 210 [°C].

**Figure 10:**
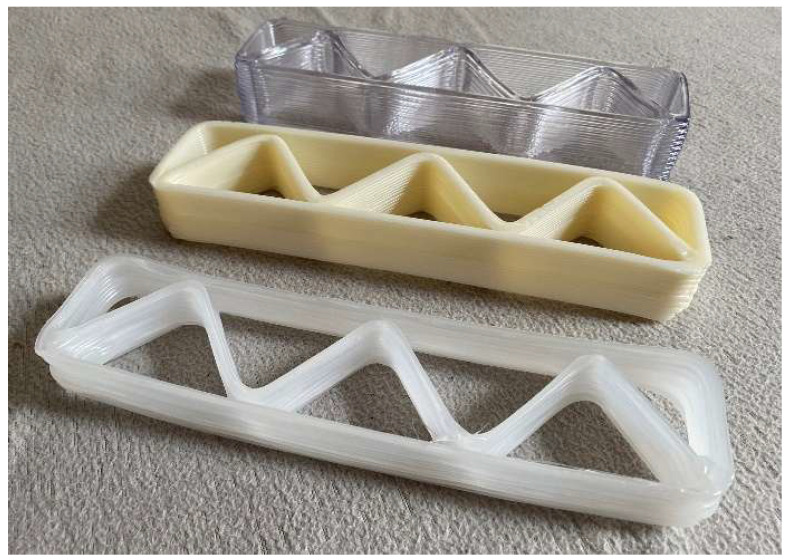
Different frameworks made of alternative materials.

To verify the possibility of modifying the manufacturing parameters, the process of printing samples of parts made of other materials was conducted (Figure 10). The values of the key process parameters are shown in Table 3. The test carried out confirmed the capabilities of the method, and the result was the correctness of the manufacturing of the parts.

**Figure 11:**
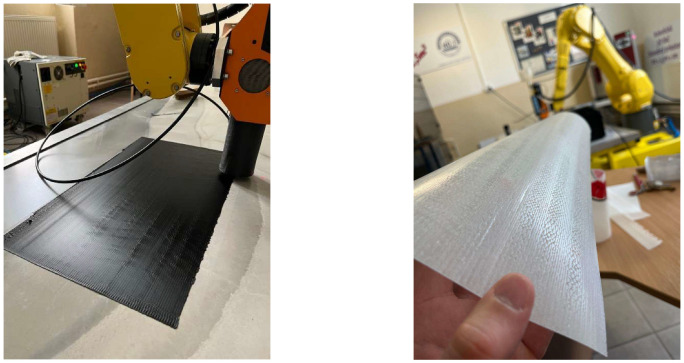
Thin mats manufactured using proposed approach.

The next step in the verification of the proposed approach was the fabrication of thin models in the form of printed mats. Changing the model parameters, the quantity of ribs was defined to achieve a consistent structure (Figure 11). To obtain an appropriate thickness, the layer value was reduced to its minimum (only one layer). The key process parameters used for printing the mats were as follows: $L_{h}$ = 0.6 [mm], $V_{rob}$ = 30 [mm/s], $T_{noz}$ = 200 [°C].

The conducted experiment was performed to demonstrate the ample capabilities of the proposed new approach in robotic 3DP processes, enabling efficient, less complex, and less time-consuming manufacturing of components.

When prototyping a production, the process often involves modifying the input geometry, for example, altering the number of ribs or the overall dimensions of the printed structure, to find suitable combinations of input and production parameters. Utilization of the proposed method reduces the time spent in the process of obtaining a new G-code by more than 80% compared to the conventional method. The time reduction achieved by the proposed method is substantiated through empirical testing. The conventional process—comprising STL model export, slicing, toolpath generation, and G-code export—typically requires around 6.5 min. In comparison, the proposed approach, which involves direct modification of the model and process parameters, reduces the total processing time to slightly over 1 min. This advantage results mainly from the ability to quickly modify the final geometry by a slight modification of the G-code without having to re-model the geometry and repeat the entire pre-process operation of exporting, importing, and generating the G-code.

### 3.5. Practical Utilization of Proposed Solution 

**Figure 12:**
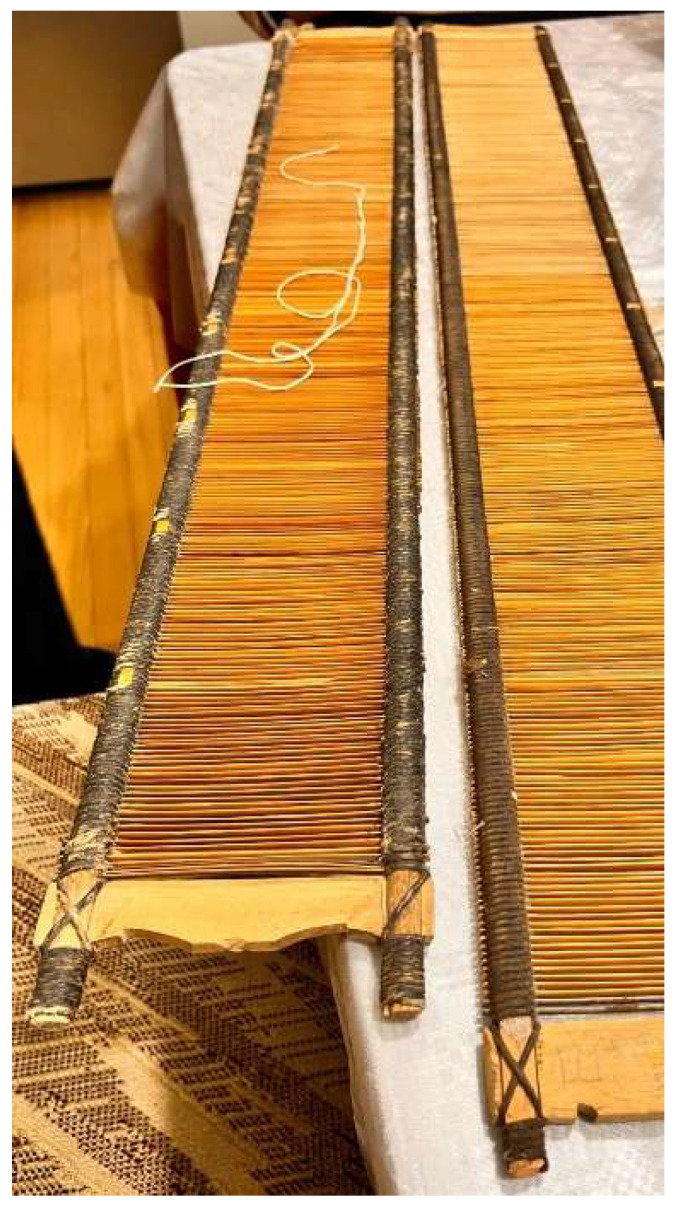
Original reed of shuttleless loom.

Positive verification of the proposed approach under laboratory conditions allowed us to begin consideration of the practical application of the method. Robotic AM was used in a reverse engineering process that aimed to reconstruct the reed of a shuttleless loom with dimensions a = 115 [mm], b = 950 [mm], h = 10 [mm] and make an alternative, more suitable part from PETG material. The original loom under consideration is shown in Figure 12.

**Figure 13:**
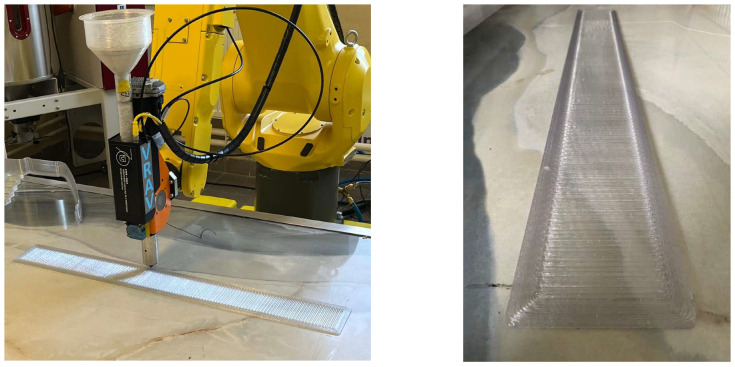
Loom reed additive manufacturing.

The reconstruction was based on detailed measurements obtained from the original part. Modifications were made to the previously developed software accordingly, so that it could be adequately reproduced using the developed method. The main reason for producing the alternative component was that the original timber material, due to its natural physical properties, had dried out and changed its dimensions over time, causing the ribs to be disproportionately spaced. In addition, the inappropriate shape of these ribs caused the carrier fibers of the fabric to be accidentally cut in the manufacturing process. In view of the manufacturer’s expanding product range, the need was expressed to produce a wider frame for the looms and to have the possibility to produce an alternative arrangement of ribs according to the actual needs. Modification of the program made it possible to generate the appropriate code and produce the part. The process of printing the reed and the final part is shown in Figure 13.

**Figure 14:**
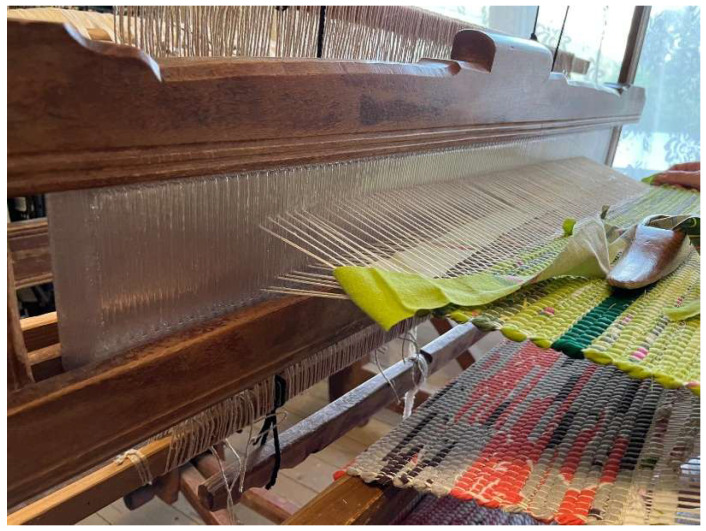
Shuttleless loom with installed reed reconstructed using proposed solution.

The manufactured element was characterized by high accuracy and adequate reproduction of the original element. Therefore, it can be concluded that the obtained element was fully functional. Consequently, it was installed in the base machine, and it fulfilled the originally intended function, as shown in Figure 14.

The practical application of the novel parametrical approach to ribbed-element slicing is further confirmation that the proposed solution is an effective and reasoned approach. This is because the reconstructed component was produced efficiently, without the need for the time-consuming process of redesigning it using various software.

## 4. Discussion

The verification of the method proposed in the article had a multi-stage character, and the main goal was to evaluate its effectiveness as well as the possibility of practical application. Each of the stages presented confirmed the validity of the use of the solution in the aspect under consideration.

The output of the developed solution enabled both the visualization of the target elements and the generation of robot control code. The obtained G-Code ensured the correct operation of the robot tool, reflecting the key paths of the manufactured model layers. Their implementation into the industrial robot made it possible to manufacture a large-size component, as well as other samples. Indeed, the proposed approach allows for the fabrication of alternative ribbed structures—both in terms of the dimensions of the manufactured part and the material. However, despite being dedicated to the manufacture of the specific parts, it is possible to modify the parameters to obtain uniform structures (as presented in the case of mats). Wider modification, in turn, allows the implementation of the method to produce structures of a similar nature. This fact highlights the applicability of the approach in real-world scenarios, where functional components can be successfully manufactured.

The main benefit of using the developed solution is the reduction in the time required to prepare the model for robotic additive manufacturing. Classically, this process is very time-consuming and requires the design of the part in a CAD environment, the subsequent generation of the STL model, and the implementation of the slicing process using well-known solutions. The proposed solution, on the other hand, makes it possible to manufacture parts in less time than the standard approach. This time will, of course, depend on the designer and operator, but the use of a purely parametric model and the definition of key process parameters will always result in a reduction in the time taken for the pre-process procedures. The proposed method has been developed for robotic, layer-by-layer planar manufacturing of components characterized by simple geometries that can be easily defined using mathematical models. Its application to complex shapes is currently limited due to the difficulty in formulating precise mathematical descriptions. Additionally, the current approach does not account for the generation of support structures, and it is not intended for non-planar slicing scenarios. Both aspects are recognized as important areas for future development and will be the subject of further research.

## 5. Conclusions

Additive manufacturing is a prevalent technology utilized in prototyping and the production of functional components. The technology is commonly associated with classical 3D printers. However, the utilization of industrial robots with multiple degrees of freedom in this process is a modern and important issue.

Since robotic 3D printing is fairly new, there are many challenges and problems associated with the process. Research is being conducted into the use of alternative printing materials, the use of multiple robots, and the fabrication of novel structures. However, a key and very important aspect of the robotic 3D printing process is the division of the model into layers, so-called ‘slicing’. In this area, researchers have proposed a variety of approaches, and one of the main problems is the lack of a dedicated solution for the direct and efficient generation of a robot’s control codes.

This paper presents a modern solution to reduce the time of pre-process procedures and improve the slicing process in robotic additive manufacturing. The proposed approach, based on a parametric model of the manufactured part, allows for some of the typical operations to be bypassed, reduces the amount of software needed, and easily generates control codes for the robot performing the printing process. The proposed solution has been verified in laboratory conditions during numerous tests, each with positive results. Thus, the conducted research confirms the validity of the mentioned approach and sets new directions in the field of robotic 3D printing.

The proposed system enhances reproducibility by enabling the consistent generation of robot control codes directly from parametric models, reducing human-dependent steps and minimizing variability. Its scalability is demonstrated through the modular slicing approach, which allows for straightforward adaptation to different robotic platforms. By reducing software dependencies and streamlining the pre-processing phase, the method contributes to improved print quality through more predictable toolpaths and optimized deposition strategies. Overall, the implementation of this solution in industrial additive manufacturing (AM) environments could significantly improve process efficiency, reduce deployment time for new parts, and support the broader adoption of robotic 3D printing in the production environment.

The limitations of the presented solution should also be noted; these are mainly due to the simplicity of the models being processed. Therefore, further research should be directed toward developing solutions for more irregular shapes and cases of objects with complex geometries. It may also be interesting to use AI techniques to generate models, directly divide them into layers, and conduct manufacturing using robotic 3D printing.

## Figures and Tables

**Table 1:** Solutions proposed in the literature.

Problem/Application	Method/Slicing Tool	References
Hybrid slicing for complex 3DP	STL-based model slicing	[1]
Sustainable wood fiber polymer printing	CAD, Cura Slicer, Robot Studio	[4]
Direct G-code for ABB robot	STL-based model slicing	[5]
Cooperative 3DP with mobile robots	Custom slicer and slicing scheme	[7,29]
Robotic AM for personalized insoles	Notepad++, MATLAB, Excel, CAD, Slic3r	[9]
Hybrid toolpath generation for 6-axis robot	CAD, MATLAB, Robot Studio	[14]
Non-planar AM for better surface and strength	C# programming, Rhino-Common API	[15]
Pattern transfer to curved surfaces	LabView, DLL	[16]
Intuitive 3D modeling via hand motion	Custom slicer	[17]
Impact of slicing on FDM quality	Multiple tools	[18]
Gradient material extrusion	ROS, Slic3r	[19]
Collision-aware code for robot	Custom contouring method	[20]
Stair-stepping in correction AM	Java-based, variable layer height	[34]
Hand motion-based slicing	Gesture-controlled slicer	[37]
Collision-free non-planar FDM slicing	Parallel slicing algorithm in Slic3r	[38]
Trajectory projection on tessellated surfaces	Mathematical algorithm	[39]
Model slicing for concrete AM	CAD-based dual-software slicing	[40]

**Table 2:** Developed script verification for different sets of basic parameters.

Set of Parameters	Result
Structure length a = 1946 [mm] Structure width b = 290 [mm] Structure height h = 240 [mm] Amount of ribs rb = 6 Layer height $L_{h}$ = 30 [mm]	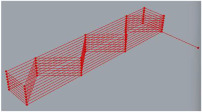
Structure length a = 1946 [mm] Structure width b = 1000 [mm] Structure height h = 240 [mm] Number of ribs rb = 10 Layer height $L_{h}$ = 30 [mm]	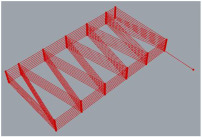
Structure length a = 3475 [mm] Structure width b = 424 [mm] Structure height h = 240 [mm] Number of ribs rb = 16 Layer height $L_{h}$ = 10.7 [mm]	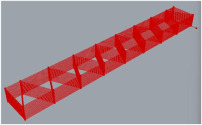
Structure length a = 3475 [mm] Structure width b = 424 [mm] Structure height h = 3000 [mm] Number of ribs rb = 16 Layer height $L_{h}$ = 22.5 [mm]	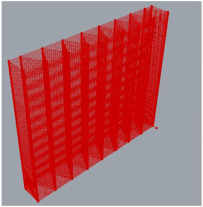

**Table 3:** Robotics 3DP process parameters and material type for samples under consideration.

Sample	Material	$L_{h}$ [mm]	$V_{rob}$ [mm/s]	$T_{noz}$ [°C]
A	PETG	2	25	215
B	ABS	1.5	20	230
C	PP	1.5	20	200

## Data Availability

Data are contained within the article. The datasets for this study are available from the corresponding author upon reasonable request.

## References

[1] Fortunato G.M., Nicoletta M., Batoni E., Vozzi G., De Maria C. (2023). A fully automatic non-planar slicing algorithm for the additive manufacturing of complex geometries. Addit. Manuf..

[2] Song Y., Yang Z., Liu Y., Deng J. (2018). Function representation based slicer for 3D printing. Comput. Aided Geom. Des..

[3] Barnett E., Gosselin C. (2015). Large-scale 3D printing with a cable-suspended robot. Addit. Manuf..

[4] Alkhatib T. (2023). Robotic 3D Printing of Sustainable Structures. Bachelor Thesis.

[5] Toshev R., Bengs D., Helo P., Zamora M. (2024). Advancing free-form fabrication: Industrial robots’ role in additive manufacturing of thermoplastics. Procedia Comput. Sci..

[6] Zhang Y., Zeng X., Gao W., Wang L., Liang X., Liu C., Yao Y., Zhang J. (2023). An Intelligent Robotic Wire Arc Additive Manufacturing System for Complex Metal Parts. Cybern. Syst..

[7] Li Z., Weng D., Chen L., Ma Y., Wang Z., Wang J. (2025). Enhanced Digital Light Processing-Based One-Step 3-Dimensional Printing of Multifunctional Magnetic Soft Robot. Cyborg Bionic Syst..

[8] Cendrero A.M., Fortunato G.M., Munoz-Guijosa J.M., De Maria C., Lantada A.D. (2021). Benefits of non-planar printing strategies towards eco-efficient 3D printing. Sustainability.

[9] Li S., Nguyen-Xuan H., Tran P. (2022). Digital design and parametric study of 3D concrete printing on non-planar surfaces. Autom. Constr..

[10] Tiryaki M.E., Zhang X., Pham Q. Printing-while-moving: A new paradigm for large-scale robotic 3D Printing. Proceedings of the International Conference on Intelligent Robots and Systems (IROS).

[11] Ondočko Š., Svetlík J., Šašala M., Bobovský Z., Stejskal T., Dobránsky J., Demeč P., Hrivniak L. (2021). Inverse Kinematics Data Adaptation to Non-Standard Modular Robotic Arm Consisting of Unique Rotational Modules. Appl. Sci..

[12] McPherson J., Bliss A., Smith F., Hariss E., Zhou W. A slicer and simulator for cooperative 3d printing. Proceedings of the Solid Freeform Fabrication 2017: The 28th Annual International Solid Freeform Fabrication Symposium—An Additive Manufacturing Conference.

[13] Tamir T.S., Xiong G., Shen Z., Leng J., Fang Q., Yang Y., Jiang J., Lodhi E., Wang F.Y. (2023). 3D printing in materials manufacturing industry: A realm of Industry 4.0. Heliyon.

[14] McPherson J., Zhou W. (2018). A chunk-based slicer for cooperative 3D printing. Rapid Prototyp. J..

[15] Pelzer L., Hopmann C. (2020). Additive manufacturing of non-planar layers with variable layer height. Addit. Manuf..

[16] Rodriguez-Padilla C., Cuan-Urquizo E., Roman-Flores A., Gordillo J.L., Vázquez-Hurtado C. (2021). Algorithm for the conformal 3D printing on non-planar tessellated surfaces: Applicability in patterns and lattices. Appl. Sci..

[17] Lee T. (2018). Hand Motion Based 3D Printing Slicer. Master’s Thesis.

[18] Nayyeri P., Zareinia K., Bougherara H. (2022). Planar and nonplanar slicing algorithms for fused deposition modeling technology: A critical review. Int. J. Adv. Manuf. Technol..

[19] Craveiro F., Bártolo H.G., Bártolo P.J., Duarte J. Fabricating construction elements with varying material composition: A case study. Proceedings of the MATADOR Conference.

[20] Werner J., Aburaia M., Raschendorfer A., Lackner M. (2021). MeshSlicer: A 3D-Printing software for printing 3D-models with a 6-axis industrial robot. Procedia CIRP.

[21] Luu Q.K., La H.M., Ho V.A. A 3-dimensional printing system using an industrial robotic arm. Proceedings of the IEEE/SICE International Symposium on System Integration.

[22] Insero F., Furlan V., Giberti H. A novel infill strategy to approach non-planar 3D-printing in 6-axis robotized FDM. Proceedings of the 2022 18th IEEE/ASME International Conference on Mechatronic and Embedded Systems and Applications (MESA).

[23] Bryła J., Martowicz A. (2021). Study on the importance of a slicer selection for the 3D printing process parameters via the investigation of G-Code readings. Machines.

[24] Piedra-Cascón W., Krishnamurthy V.R., Att W., Revilla-León M. (2021). 3D printing parameters, supporting structures, slicing, and post-processing procedures of vat-polymerization additive manufacturing technologies: A narrative review. J. Dent..

[25] Hong Q., Lin L., Li Q., Jiang Z., Fang J., Wang B., Liu K., Wu Q., Huang C. (2021). A direct slicing technique for the 3D printing of implicitly represented medical models. Comput. Biol. Med..

[26] Kristiawan R.B., Imaduddin F., Ariawan D., Ubaidillah N., Arifin Z. (2021). A review on the fused deposition modeling (FDM) 3D printing: Filament processing, materials, and printing parameters. Open Eng..

[27] Pires J.D.A.C. (2021). Industrial Robot Based 3D printer. Master’s Thesis.

[28] Bhooshan S., Ladinig J., Van Mele T., Block P. (2018). Function representation for robotic 3D printed concrete. Robotic Fabrication in Architecture, Art and Design 2018.

[29] Yu L., Huang Y., Zhongyuan L., Xiao S., Liu L., Song G., Wang Y. Highly informed robotic 3D printed polygon mesh: A nobel strategy of 3D spatial printing. Proceedings of the 36th Annual Conference of the Association for Computer Aided Design in Architecture (ACADIA).

[30] Wüthrich M., Gubser M., Elspass W.J., Jaeger C. (2021). A novel slicing strategy to print overhangs without support material. Appl. Sci..

[31] Li X., Liu W., Hu Z., He C., Ding J., Chen W., Wang S., Dong W. (2024). Supportless 3D-printing of non-planar thin-walled structures with the multi-axis screw-extrusion additive manufacturing system. Mater. Des..

[32] Xu Z., Song T., Guo S., Peng J., Zeng L., Zhu M. (2021). Robotics technologies aided for 3D printing in construction: A review. Int. J. Adv. Manuf. Technol..

[33] Ariffin M.K.A.M., Sukindar N.A., Baharudin B.H.T., Jaafar C.N.A., Ismail M.I.S. (2018). Slicer method comparison using open-source 3D printer. IOP Conf. Ser. Earth Environ. Sci..

[34] Eyercioğlu Ö., Aladağ M. (2021). Non-planar toolpath for large scale additive manufacturing. Int. J. 3D Print. Technol. Digit. Ind..

[35] Menges A., Sheil B., Glynn R., Skavara M. (2017). Infundibuliforms: Kinetic Systems, Additive Manufacturing for Cable Nets and Tensile Surface Control. Fabricate 2017.

[36] Nycz A., Noakes M.W., Masuo C.J., Love L.J. Control system framework for using g-code-based 3d printing paths on a multi-degree of freedom robotic arm. Proceedings of the Solid Freeform Fabrication 2018: The 29th Annual International Solid Freeform Fabrication Symposium—An Additive Manufacturing Conference, SFF 2018.

[37] Senthil T.S., Vel N.R.O., Puviyarasan M., Babu S.R., Surakasi R., Sampath B. (2023). Industrial robot-integrated fused deposition modelling for the 3D printing process. Development, Properties, and Industrial Applications of 3D Printed Polymer Composites.

[38] Ahlers D., Wasserfall F., Hendrich N., Zhang J. 3D printing of nonplanar layers for smooth surface generation. Proceedings of the IEEE 15th International Conference on Automation Science and Engineering (CASE).

[39] Bhatt P.M., Malhan R.K., Rajendran P., Gupta S.K. (2020). Building free-form thin shell parts using supportless extrusion-based additive manufacturing. Addit. Manuf..

[40] Lazarev Y., Krotov O., Belyaeva S., Petrochenko M. (2020). 3D environmentally friendly concrete printing model preparation. E3S Web Conf..

[41] Zhang X., Li M., Lim J.H., Weng Y., Tay Y.W.D., Pham H., Pham Q. (2018). Large-scale 3D printing by a team of mobile robots. Autom. Constr..

[42] Puzatova A., Shakor P., Laghi V., Dmitrieva M. (2022). Large-scale 3D printing for construction application by means of robotic arm and gantry 3D printer: A review. Buildings.

[43] Kulikov A.A., Sidorova A.V., Balanovskii A.E. (2021). Programming industrial robots for wire arc additive manufacturing. Proceedings of the 6th International Conference on Industrial Engineering (ICIE 2020).

[44] Xiao J., Ji G., Zhang Y., Ma G., Mechtcherine V., Pan J., Wang L., Ding T., Duan Z., Du S. (2021). Large-scale 3D printing concrete technology: Current status and future opportunities. Cem. Concr. Compos..

[45] Insero F., Furlan V., Giberti H. (2025). Non-planar slicing for filled free-form geometries in robot-based FDM. J. Intell. Manuf..

